# Molecules, Up Your Spins!

**DOI:** 10.3390/molecules29081821

**Published:** 2024-04-17

**Authors:** Danila A. Barskiy

**Affiliations:** 1 Institut für Physik, Johannes-Gutenberg-Universität Mainz, 55128 Mainz, Germany; dbarskiy@uni-mainz.de; 2 Helmholtz Institut Mainz, 55128 Mainz, Germany; 3 GSI Helmholtzzentrum für Schwerionenforschung, 64291 Darmstadt, Germany

Nuclear magnetic resonance (NMR) spectroscopy and magnetic resonance imaging (MRI) are indispensable tools in science and medicine, offering insights into the functions of biological processes. Traditional analytical methods, such as mass spectroscopy and chromatography, are significantly more sensitive, but they require the destruction of samples during analysis [1,2,3]. In contrast, NMR and MRI rely on the detection of signals from nuclear spins without altering samples, making these modalities truly non-invasive and ideal for studying biological systems.

The non-invasiveness of NMR/MRI stems from the low energy of spin-field interaction quanta (hν) compared to the thermal energy of the environment (kT). Here, $\nu =\left|\gamma B\right|$ is a frequency of spin precession (γ is a gyromagnetic ratio of the spins, and B is a static magnetic field used in NMR/MRI, typically several tesla), T is temperature, and h and *k* are Plank’s and Boltzmann’s constants, respectively. In magnetic resonance, *polarization* (*P*) is defined as a dimensionless quantity directly proportional to the ratio of the above-mentioned energies [4], P~hν/kT. At room temperature, kT is about $5\;\cdot \;10^{-21}$ joules, while hν for protons is on the order of $10^{-25}$ joules even at the magnetic fields of modern high-field NMR spectrometers. It is this low interaction energy between spins and the external field that is a cornerstone to the noninvasiveness of NMR/MRI; spins report on their environment without disturbing it [5,6]. At the same time, low spin-field interaction energy dictates the overall poor sensitivity of magnetic resonance techniques. Given typical *p* values of 0.001–0.0001%, only relatively large concentrations of spins (>1 mM) can be measured with sufficient signal-to-noise ratio (SNR) in a conceivable amount of time [7,8,9]. This sets stringent limits on the applications of NMR/MRI and constrains studies to large sample volumes as compared to other analytical methods.

*Hyperpolarization* refers to situations in which *P* can be much higher than a thermal equilibrium value, for example, >10% [10,11]. By enhancing polarization levels through hyperpolarization techniques (see below), NMR/MRI can achieve sensitivity to enable detection of low-concentration samples (e.g., <1 μM) with high SNR. As an example of hyperpolarization, see Figure 1. At the top, an ^15^N NMR spectrum of a fully labeled ^15^N-pyridine as a neat liquid was measured at a magnetic field of 9.4 tesla. Since molecules are isotopically enriched with ^15^N nuclei (>99%) and present at high concentrations (~12.4 M), a sufficient SNR is obtained in a single acquisition. For comparison, a sample of 50 mM metronidazole—a well-known antibiotic and hypoxia probe—at a natural isotopic abundance of ^15^N (0.35%) gives a strong ^15^N NMR signal after being flushed with parahydrogen (*p*H_2_) gas for ~30 s (Figure 1, bottom). For the same sample at thermal equilibrium to give an NMR signal with SNR comparable to the neat, [^15^N]-labeled pyridine, about 30 years of continuous signal averaging would be necessary. This example demonstrates the power of hyperpolarization, in this case, the SABRE technique (SABRE = signal amplification by reversible exchange): molecules at low concentration and natural isotopic abundance can be detected with sufficient SNR on a time scale of seconds [12].

This *Editorial essay* briefly explores applications of hyperpolarization techniques in both medical diagnostics and emerging spin technologies. The current landscape of methods is examined, with a particular focus on medical applications. Hyperpolarization-enhanced MRI is compared to positron-emission tomography (PET), and the promises in molecular imaging and disease monitoring are critically assessed. I also explore novel quantum sensing modalities empowered by spin hyperpolarization in biomedical research and beyond. Through this analysis, I hope to highlight promising research directions related to spin technologies that may refine our understanding of key (bio)molecular processes.

**Hyperpolarization Techniques**

Given the wealth of existing literature on the topic of nuclear and electron hyperpolarization, here I refrain from delving into the foundational principles of these techniques. However, it is important to note that hyperpolarization technologies broadly fall into two categories, although some methods may formally qualify for both groups:

*Techniques Utilizing Electromagnetic Fields*

A significant fraction of hyperpolarization methods uses electromagnetic fields. Notably, in dynamic nuclear polarization (DNP), polarization transfer from unpaired electrons to spin-active nuclei is facilitated by the application of microwaves [14,15,16,17,18,19]. DNP allows for the generation of strong NMR signals for molecules that increase by orders of magnitude compared to thermal equilibrium, resulting in dramatically decreased signal averaging times. DNP encompasses various methodologies such as Overhauser-DNP and dissolution-DNP, as well as emerging approaches like bullet-DNP [18,19,20]. Optical pumping techniques involving visible or infrared radiation play a major role in the hyperpolarization of both electron and nuclear spins. Spin-exchange optical pumping (SEOP) and metastability-exchange optical pumping (MEOP) of noble gases [21] exemplify this group of methods, along with optical pumping of defects in solids like NV-centers in diamond [22,23,24]. Visible light applied for generating hyperpolarization is a signature of chemically induced dynamic nuclear polarization (CIDNP) and related approaches [5,25,26].

In principle, pumping with electromagnetic radiation allows polarizing molecules in all phases of ordinary matter (gas, liquid, solid, and even plasma [27]). Direct pumping of nuclear magnetization with light seems to be possible in the gas or solid via optical pumping, while hyperpolarization of molecules in solution necessitates more complex interactions.

*Techniques Utilizing Chemistry and Spin Statistics*

Another subset of hyperpolarization techniques relies on intricate spin statistics to facilitate the generation of hyperpolarized states. Chemical reactions and chemical exchange, notably in parahydrogen-induced polarization (PHIP), underpin these methodologies. PHIP variants like PASADENA, ALTADENA, and SABRE demonstrate remarkable polarization levels (above 50%) on various nuclei [14,28]. While challenges remain in clinical translation, specifically the ability to control all stages of chemical transformations and fields at each moment of sample transfer, recent advancements have showcased reproducible polarization levels on biologically relevant nuclei, fostering optimism for future developments [29,30,31].

For comprehensive exploration of hyperpolarization, readers are encouraged to consult recent reviews that delve into the physicochemical principles of these techniques [32]. The semantic breadth of “hyperpolarization” terminology highlights its diverse manifestations, which extend beyond simple magnetization to encompass complex spin orders with broad implications for both fundamental research and technological innovation [10,33,34].

**Medical Applications of Hyperpolarization**

As of 2024, biomedical science remains a major driver for hyperpolarization research, as evidenced by the number of peer-reviewed publications devoted to this subject in recent years [35,36,37]. The interest is not surprising due to the immense applicability of NMR/MRI in medical diagnostics, even without using hyperpolarization. While there are no yet clear avenues for generating hyperpolarization inside a living object without bringing hyperpolarized molecules from the outside (exogenous injections do not make hyperpolarization-enhanced MRI fully non-invasive), the existing alternative clinical approaches to monitoring metabolism involve radioactive samples, and, thus, MRI is freed from this complication. Coupled with the ability to select specific regions in the object under study and harness information from heteronuclei (^13^C, ^15^N, ^129^Xe, etc.), hyperpolarization-enhanced MRI provides a novel toolkit for understanding the chemical composition and functions of tissue, disease progression, and treatment [38,39,40].

Conceptually, in the context of *molecular imaging* (i.e., imaging of specific molecules and their transformations rather than imaging of bulk medium), hyperpolarization-enhanced MRI shares similarities with positron-emission tomography (PET), and it is worth delving deeper into the comparative analysis of these two modalities. Both technologies, in their current implementation, require the injection of exogenous contrast agents bearing a signal-generating nuclear isotope.

*Comparison of PET and Hyperpolarization-Enhanced MRI*

In PET, a radioactive agent is injected into the patient (ideally) immediately after its production. Radioactive decay typically happens on a timescale of minutes (*τ*_1/2_~110 min for ^18^F nuclei), generating positrons that annihilate with the nearby matter; measured signals are derived from the detection of γ-photons emitted upon this annihilation [41]. While PET offers high imaging sensitivity, its resolution is limited to 3–5 mm [42]. 

In hyperpolarization-enhanced MRI, a hyperpolarized exogenous contrast agent (with polarization typically “stored” in the magnetization of heteronuclei such as ^13^C) has a short in vivo lifetime, providing a time window of, at best, up to 5 min after injection. This can be used for angiography and perfusion (Figure 2) but is often not sufficient for monitoring metabolic processes of interest [43]. A time window of at least a few hours would be more appropriate for studying unknown details of the Krebs cycle (such as its reversibility) and other metabolic transformations [44]. However, unlike PET, MRI faces no fundamental resolution limitations, with bottlenecks being practical, e.g., available SNR per voxel, ability to provide large field gradients in short time intervals, etc. The typical resolution of conventional proton MRI is about 1 mm, and sub-100-micrometer-resolution microimaging has been demonstrated with hyperpolarization [45].

Despite these differences, both hyperpolarization-enhanced MRI and PET can visualize specific metabolites by using tracer molecules and appropriate image reconstruction techniques. In MRI, [^13^C]-pyruvate is one of the most promising and well-developed hyperpolarized contrast agents for observing metabolism within the Krebs cycle [38], while PET-agents [^18^F]-FDG (fluorodeoxyglucose) and [^13^N]-ammonia are routinely used clinically (and numerous other agents have been tried in research [46]).

**Figure 2:**
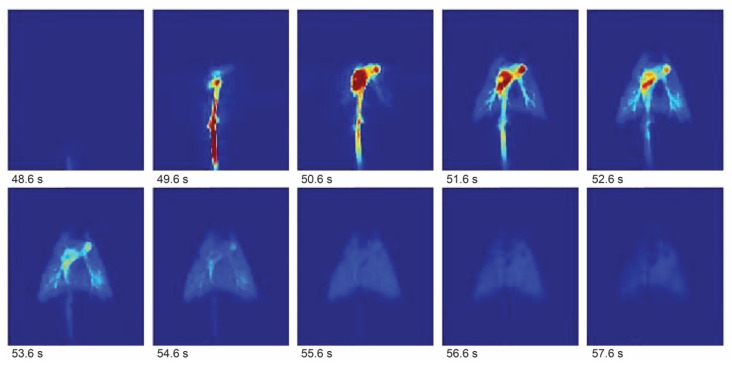
Hyperpolarization-enhanced ^13^C magnetic resonance images showing the lungs of a pig after injection of hyperpolarized [^13^C]-2-hydroxyethylacrylate with 1 s time resolution. Adapted with permission from [47].

It is interesting to note that both PET and hyperpolarization-enhanced heteronuclear MRI exploit the lack of intrinsic background signal to observe molecular processes without interference. PET has no background signal owing to the absence of radioactive positron-emitting nuclei in the body as well as a quiet gamma-ray background in the environment. Similarly, signals from naturally abundant thermally polarized heteronuclei such as ^13^C or ^15^N are virtually absent in MRI. Monitoring metabolic changes by conventional ^1^H MRI, on the other hand, is challenging due to the large background signal originating from thermally polarized protons in H_2_O and lipids. Stargazing provides the following good analogy: observation of stars from inside a megapolis is challenging because of optical pollution; one would need to go to the mountains or far in the wilderness (where the background light is absent) to notice myriads of stars with a bear eye. One should note that there have been notable advancements in molecular imaging with the utilization of perdeuterated exogenous contrast agents [48,49]. Unlike hyperpolarization, this technique does not enhance polarization beyond its thermal value but, instead, relies on deuterium—a stable isotope with low natural abundance—to discern chemicals in vivo. Further exploration could potentially involve other nuclei with rapid relaxation times engaged in biologically significant chemistry [50].

Developing endogenous hyperpolarized contrast agents generated on demand (or naturally produced) inside the object that is being investigated seems highly desirable. Green fluorescent protein (GFP) in combination with optical detection serves as an inspiration: generation of GFP is possible in various environments via genetic manipulations [51]. In the case of MRI, approaches to generating genetically encoded signal contrast in vivo have been proposed based on the use of hyperpolarized ^129^Xe gas [52,53]. Parahydrogen is another option since it offers a unique possibility of bringing latent nuclear spin order inside the object to be studied in such a way that magnetization is generated only in vivo and on demand (Figure 3) [54]. While typically information encoding and signal detection are inseparable parts of the measurement, MRI fundamentally permits separating these two steps in time and/or space. Further interdisciplinary innovation is likely necessary to unlock opportunities provided by genetically encoded hyperpolarized MRI sensors [55].

*Challenges of Hyperpolarized Molecular MRI*

Despite its immense potential, the widespread clinical adoption of hyperpolarization-enhanced MRI faces significant constraints. These limitations primarily stem from the prevalence of hardware optimized for detecting protons (^1^H) and the absence of refined pulse sequences for effective polarization transfer, crucial for improving the SNR of heteronuclear signals. Additionally, hyperpolarization-enhanced NMR/MRI necessitates interdisciplinary working groups and requires advanced infrastructure [56].

The first proposals for using hyperpolarized contrast agents emerged in the early 1990s, but the steady stream of research publications has (up to date) not been sufficient to convince practicing physicians of their utility. As of 2024, the number of hospitals in the world equipped with the necessary devices and expertise to observe metabolic transformations using hyperpolarization-enhanced ^13^C MRI remains fewer than 20 [56]. While the principles of the *d*DNP methodology have been known since 2003 [19], the anticipated widespread clinical application of this method has not materialized, despite advancements in other research areas driven by Moore’s Law [57]. Polarization levels are not universally high, even for *d*DNP, and can vary depending on the specific preparation method employed. They are also extremely technically challenging to maintain. However, potentially the biggest drawback of the existing modality is the short lifetime of hyperpolarized molecules in vivo—particularly concerning the most interesting molecules like pyruvate (*T*_1_ of carbon-13 at 3 T is only ~30 s in vivo [58])—limiting clinical applications to tissues with high cellularity and rapid transfer through cell membranes. It is essential for the research community to maintain a balanced perspective on this emerging technology since even niche applications without revolutionary clinical impact can still be valuable.

In summary, hyperpolarization-enhanced MRI is a molecular imaging modality offering sensitivity and resolution comparable to PET yet enabling unique chemical specificity. However, resolving challenges related to the polarization lifetime is critical for successful clinical adaptation. As hyperpolarization technology matures, the prospect of MRI with heteronuclear detection becoming commonplace holds promise for advancing our understanding of metabolic changes in both research and clinical contexts. Moreover, the development of joint modalities combining the sensitivity of PET with the resolution of MRI could further enhance diagnostic capabilities, signaling exciting prospects for future developments.

**Emerging Spin Technologies**

In today’s world, appreciation of technology may often outweigh appreciation for the research that underpins it. Nevertheless, it is crucial to recognize that without ongoing scientific exploration, technological innovation would likely stagnate. This is particularly evident in the realm of spin technologies, where the fundamental quantum nature of spins opens doors to a plethora of applications across diverse fields [59]. From controlling chemical reactions dependent on nuclear spins to the development of quantum sensors utilizing single defects in crystal lattices, the breadth of potential applications is vast [60].

In the context of MRI, quantum phenomena such as entanglement and long-lived spin states offer avenues for extending polarization lifetimes, thus enhancing imaging capabilities [61]. PHIP, SABRE, and, in general, magnetization transfer catalysis (MTC) demonstrate how transient molecular interactions can be leveraged to amplify spin signals, enabling novel detection schemes. In addition to hyperpolarization, the principles of quantum metrology hold potential for enabling precise differentiation of chemicals and their transformations through high-resolution analysis of spectral frequencies and phases [59]. By leveraging key quantum concepts like squeezing and entanglement, spin techniques could improve MRI by achieving unprecedented resolution.

Hyperpolarization, beyond its application in MRI, holds promising potential for analytical chemistry [9,12], particularly in contexts where hyperpolarization and detection can be achieved without reliance on costly equipment. This prospect could democratize NMR and unlock its vast analytical capabilities for developing countries.

**Conclusions**

In the landscape of hyperpolarization-enhanced NMR/MRI, challenges and opportunities lie ahead. The following key questions persist: Will nuclear hyperpolarization unveil novel, previously unknown dimensions of metabolism? How can spin order be efficiently preserved in molecules within biochemical processes over extended timeframes beyond a few minutes? Will innovative NMR detection methods, endowed by hyperpolarization, transition to practical clinical applications? Could portable point-of-care NMR devices revolutionize healthcare diagnostics?

These questions not only underscore the ongoing evolution of hyperpolarization techniques but also point to potential avenues for future research and technological advancement. It is already evident that hyperpolarization represents a promising trajectory—one that complements established high-field NMR/MRI modalities—in our journey to improve magnetic resonance methods. The rallying cry remains, “Molecules, up your spins!” and with each discovery, we shape the future of truly quantum molecular imaging.

## Figures and Tables

**Figure 1:**
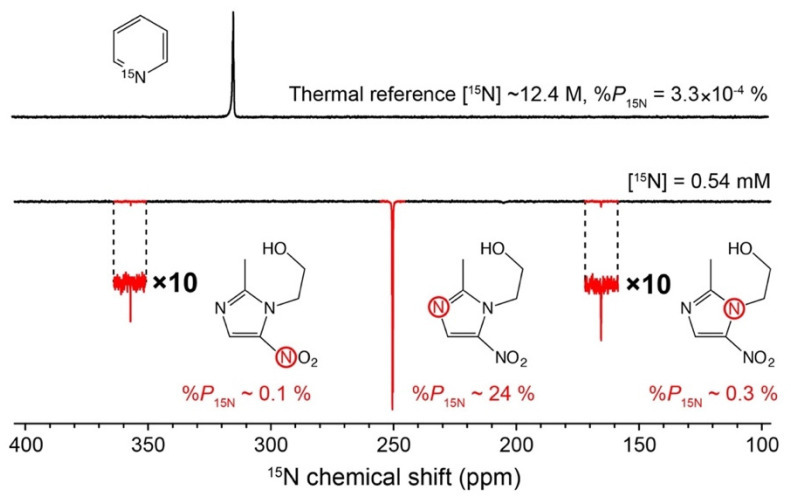
Example of hyperpolarization. (**Top**) A typical ^15^N NMR spectrum of ^15^N-pyridine (~99% ^15^N labeling) recorded at 9.4 tesla. (**Bottom**) SABRE-enhanced ^15^N NMR spectrum of metronidazole at 150 mM (^15^N nuclei at natural isotopic abundance of 0.35%) after 30 s of parahydrogen bubbling (50% *p*H_2_ enrichment fraction). Nitrogen-15 in the -NO_2_ group is demonstrated to have a *T*_1_ of ~15 min. Reproduced with permission from [13].

**Figure 3:**
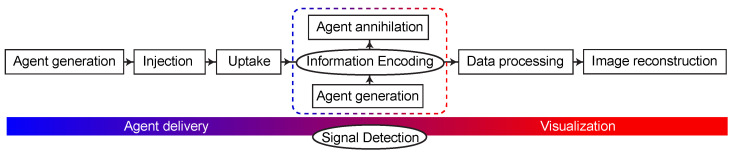
Stages of molecular imaging using exogenous and endogenous (dashed line) contrast agents.
